# Effectiveness of intermittent cash incentives to increase step counts

**DOI:** 10.1002/jaba.2929

**Published:** 2024-12-27

**Authors:** Sydney R. Batchelder, Amanda Devoto, Wendy Donlin Washington

**Affiliations:** ^1^ Department of Psychology University of North Carolina Wilmington Wilmington NC USA; ^2^ Department of Psychology Eastern Michigan University Ypsilanti MI USA

**Keywords:** contingency management, financial incentive, physical activity, prize bowl

## Abstract

Only 25% of adults meet both aerobic and strength training recommendations for physical activity. Contingency management interventions have been used to increase physical activity; however, they may be cost prohibitive. Intermittently provided incentives lower costs and are effective for various health behaviors. The present study investigated whether intermittent cash incentives can increase physical activity (step counts). The researchers used a reversal design with 21 participants, and goals during the intervention were set using a percentile schedule. Contingent on meeting goals, participants could earn the opportunity to draw tickets that corresponded to either no cash or cash incentives. Step counts significantly increased from baseline to the intervention phase. Overall, intermittent cash incentives may be a viable and cost‐effective approach to promoting health behavior.

Physical activity is defined as bodily movement that results in energy expenditure; different movements result in different levels of energy expenditure (Centers for Disease Control and Prevention, 2018). Physical activity levels vary from sedentary (e.g., sitting), to light (e.g., slow walking), moderate (e.g., walking), and vigorous (e.g., running). Many American adults do not meet the recommended levels of physical activity. Physical activity guidelines have evolved from focusing on continuous vigorous activity to moderate activity, including daily activities like walking (Ding et al., 2020). Studies show that taking more steps per day is associated with reduced risk of all‐cause mortality (Paluch et al., 2021, 2022; Saint‐Maurice et al., 2020). For example, physical inactivity is linked to obesity (Silveira et al., 2022), whereas higher step counts are associated with reduced risk of cardiovascular disease and cancer mortality (Saint‐Maurice et al., 2020). Physical activity is also associated with reduced depressive (Heissel et al., 2023) and anxiety symptoms (Carter et al., 2021). Current guidelines recommend 150 min of moderate‐intensity activity per week, which is equivalent to walking 20 min per day (Centers for Disease Control and Prevention, 2018).

Effective interventions to increase physical activity include antecedent interventions and consequence interventions. Antecedent interventions are any manipulation of environmental conditions prior to the behavior (Smith, 2011). In the physical activity literature, common antecedent interventions include goal setting, self‐monitoring, and prompts, whereas consequence interventions are those that provide a consequence contingent on meeting a goal (Rotta et al., 2023). Contingency management (CM) is a consequence‐based intervention wherein “incentives are provided contingent on objective evidence of behavior change”  (Davis et al., 2016, p. 1). CM interventions are among the most effective treatments for health behaviors such as drug use (e.g., Higgins et al., 2007), smoking (e.g., Jarvis & Dallery, 2017), and physical inactivity (e.g., Washington et al., 2014).

CM is based on differential reinforcement. Research concerning differential reinforcement has demonstrated that higher magnitude incentives that are delivered more immediately, more frequently, and in the absence of competing reinforcers are most likely to be effective in changing behavior (e.g., Mace et al., 1994; Neef et al., 1992, 1993, 1994; Saini et al., 2021). The effect of magnitude has also been demonstrated in the CM literature (Dallery et al., 2001; Silverman et al., 1999). However, interventions that maximize frequency and magnitude of incentives can be expensive and effortful to implement (Petry et al., 2004). Therefore, reducing cost and effort barriers for implementation can potentially enhance the dissemination of these behavior change techniques. Using different types of incentives can affect both cost and ease of implementation. For example, prior CM research has evaluated the efficacy of cash, vouchers, and prizes as incentives. Cash incentives include cash and electronic monetary rewards. Vouchers are exchangeable for goods and services, whereas in a prize system, draws of tickets placed in a bowl provide the opportunity to earn tangible prizes (e.g., stickers, T‐shirt, Kindle) of either no value, small (e.g., $1), medium (e.g., $20), or large (e.g., $50–$100) values.

The efficacy of incentive type (e.g., cash, vouchers, or prizes) has been mixed in prior research. Festinger et al. (2014) found that vouchers and cash incentives were equally effective at increasing abstinence for cocaine and attendance relative to a control group. However, Vandrey et al. (2007) evaluated continuous vouchers versus continuous cash incentives of various amounts. At higher values ($50 and $100 incentives), the cash incentives but not the vouchers resulted in greater cocaine abstinence relative to the control condition. Overall, this suggests that manipulating the type of incentive may affect the efficacy of incentives as an intervention.

In addition to the type of incentive, the probability of the incentive can also vary. Incentives may be delivered continuously (i.e., according to a fixed‐ratio [FR] 1 reinforcement schedule), where each response results in an incentive, or intermittently, where each response has a specified probability of producing the incentive. Intermittent incentives are often used in a prize system, where the individual has a lower probability of receiving a high‐magnitude tangible prize than a lower magnitude tangible prize. Petry et al. (2000) used intermittent tangible prize incentives to increase alcohol abstinence in which participants earned a draw for every negative breath alcohol sample. Participants chose tickets of paper from a bowl; 25% of tickets included textual praise, 68% included small tangible prizes worth $1, and the remaining 7% included either medium (worth $20) or large (worth $50) tangible prizes. This study demonstrated the efficacy of an intermittent, low‐cost method for reducing relapse. Comparisons of continuous and intermittent incentives have shown that they are similarly efficacious in altering behavior (Petry et al., 2005, 2007, 2016). Petry et al. (2016) compared the influence of intermittent versus continuous incentives on methadone treatment maintenance among individuals with cocaine use disorder. The results indicated that intermittent incentives performed as well as continuous incentives in supporting cocaine abstinence. The generality of this approach has been demonstrated in subsequent research to address drug use among individuals with cocaine use disorder and opioid use disorder (Petry et al., 2005, 2007, 2016).

The use of intermittent incentive arrangements to promote physical activity has been less extensive than for drug abstinence. In two relevant studies, intermittent tangible prize incentives increased the intensity of physical activity during interval training for participants with developmental disabilities (May & Treadwell, 2020) and daily step counts among typically developing adults via a smartphone application intervention (Patel et al., 2018). In addition, Washington et al. (2014) used intermittent tangible prize incentives to increase daily steps for typically developing adults. For this study, the researchers determined the criteria for reinforcement using a combination of goal setting and a percentile schedule of reinforcement. In goal setting, the researcher specifies the criterion for reinforcement prior to the occurrence of the behavior (Fellner & Sulzer‐Azaroff, 1984). Goal setting is effective in cases where goals are achievable and feedback is received about progress toward the goal (Locke & Latham, 2002). Although goal setting is often an effective strategy for behavior change, the lack of consequences associated with the altered behavior results in poor generalization over time (Choi & Johnson, 2022; Fellner & Sulzer‐Azaroff, 1984; Jessup & Stahleski, 1999).

Washington et al. (2014) combined goal setting with a percentile schedule of reinforcement. Percentile schedule arrangements are determined based on the following equation:

(1) $$k=\left(m+1\right)\left(1-w\right).$$

In this equation, *k* is the rank of response that must be exceeded to receive reinforcement, *m* is the number of prior observations, and *w* is the probability that the response will receive reinforcement and is specified by the researcher (Galbicka, 1994). For example, if *m* is set to five prior observations and *w* is to set to 0.5, then solving for *w* according to Equation 1 would yield *k* = (5 + 1) (1 – 0.5), or *k* = 3. In this example, *k* of 3 denotes the observation ranked third among the five most recently observed and ranked observations is the value the current observation must exceed to meet the criterion for reinforcement. Percentile schedules are useful for setting goal criteria because the probability (*w*) and prior observations (*m*) can be customized based on the participant's needs and performance.

Washington et al. (2014) used a percentile schedule of reinforcement whereby participants needed to walk more steps than the response ranked 3rd (*w* ~ 0.7) of the last 7 observations (*m* = 7) to receive a ticket draw. Ticket draws resulted in 50% textual praise, 42% small tangible prizes, and 8% medium/large tangible prizes. Of the 11 participants in the study, four individuals (36%) improved their step counts during the intervention. These results are less robust than those found in other studies of the influence of intermittent tangible prize incentives on physical activity (May & Treadwell, 2020; Patel et al., 2018) and substance use disorder (Petry et al., 2000, 2016).

We hypothesize that the results of Washington et al. (2014) were less robust than those from other intermittent incentive arrangements because of several factors. First, Washington et al. had no ceiling for participant performance during baseline; two participants in the study had mean step counts above 10,000 steps during baseline, making it difficult to show improvement during the course of the study. Second, the tangible prizes available to choose from may not have been of sufficient value to maintain their behavior. It is possible that tangible prizes were less effective relative to cash incentives (e.g., Vandrey et al., 2007). To improve on the methods of Washington et al., the present study excluded participants whose mean step counts during baseline exceeded 10,000 steps, eliminating any ceiling effect. In addition, the present study used intermittent cash incentives instead of the tangible prizes available in Washington et al. to improve daily step counts.

## METHOD

### Participants

Adults (aged 18–65) were recruited through flyers posted at a university in the southeastern United States. Eligibility required that participants were not pregnant, did not have asthma, engaged in less than 10,000 steps per day on average during baseline, and indicated they could participate in physical activity. Table 1 includes demographic information for all participants who were screened for the study. Twenty‐one eligible participants who ranged in age from 18 to 60 completed the study.

**TABLE 1:** Baseline characteristics.

Participant	Sex	Age	BMI	% Body fat	Reason dropped
301	Woman	38	43.06	46	‐
302	Woman	19	19.20	19	‐
303	Woman	40	26.30	35	‐
304	Woman	41	27.32	40	‐
305	Woman	47	22.05	17	Withdrew
306	Woman	18	30.07	34	Broke Fitbit
307	Woman	47	27.69	36	‐
308	Woman	60	30.18	40	‐
309	Woman	21	32.42	37	Sick
310	Woman	22	30.29	34	‐
311	Woman	18	22.62	20	>10,000
312	Woman	18	23.60	25	Withdrew
313	Woman	21	25.90	27	‐
314	Woman	21	27.10	30	>10,000
315	Woman	18	19.78	20	Withdrew
316	Man	18	29.25	20	>10,000
317	Woman	21	20.30	14	‐
318	Woman	19	28.47	32	>10,000
319	Woman	18	30.37	35	>10,000
320	Man	18	21.31	‐	‐
321	Man	20	26.62	19	>10,000
322	Woman	21	18.20	18	Withdrew
323	Woman	18	20.79	23	>10,000
324	Woman	19	24.54	30	‐
325	Woman	22	25.30	29	‐
326	Woman	20	21.98	18	Withdrew
327	Woman	22	28.53	33	‐
328	Woman	19	18.48	15	‐
329	Woman	56	45.63	‐	‐
330	Man	22	27.00	14	‐
331	Woman	19	23.03	24	Technical problems
332	Man	19	19.67	11	‐
333	Man	19	26.22	17	‐
334	Woman	19	24.05	25	>10,000
335	Man	19	19.98	12	‐
336	Man	18	22.10	15	>10,000
337	Woman	24	27.98	33	‐
338	Woman	21	25.37	29	‐
339	Woman	22	23.66	26	>10,000
340	Woman	18	17.20	17	Sick
341	Woman	20	27.03	28	‐

*Note*: BMI = body mass index.

### Materials

#### Accelerometer

The Fitbit Ultra or Fitbit Zip tracked steps per minute throughout the day. The Fitbit Ultra required charging on a docking station that was attached via USB to a laboratory computer. The Fitbit Zip used a replaceable 3 V coin battery that lasted 3–6 months. Both the Fitbit Ultra (Dontj et al., 2015; Evenson et al., 2015) and the Fitbit Zip (Evenson et al., 2015; Ferguson et al., 2015) are valid and reliable devices for measuring physical activity.The Fitbit Zip wirelessly synced to Fitbit.com when participants were close to a computer with a wireless sync dongle (i.e., when they came to the lab). The Fitbit Ultra synced to Fitbit.com when participants came to the lab and placed their Fitbit on the docking station attached to the computer. Participants were restricted from accessing Fitbit.com to view their data and not permitted to access other components of the website (e.g., graphs of their data). Only the researchers had access to the website to verify participant step count reports.

#### Activity guide

During intake, participants were given a copy of the *Physical Activity Guidelines* issued by theDepartment of Health and Human Services (2008).

### Dependent variables

The primary dependent variable was daily step count as measured by the Fitbit accelerometers and verified by researchers via Fitbit.com. Other dependent variables included the percentage of goals met, which was calculated based on the number of days the participant exceeded their step goal divided by the total number of days during the intervention period. We also calculated the total amount of money that was earned by adding up the money earned at each ticket draw throughout the intervention.

### Procedure

The current study used an ABA reversal design in which the baselines (A) lasted 1 week each and the intervention (B) lasted 3 weeks, totaling 5 weeks of participation. Participants were instructed to wear the Fitbit during the baseline, but no step criteria or goals were introduced. During the intervention phase, the researchers set individualized step goals for participants (see Establishing step criteria section below). If participants met the daily goal, they could earn money through an intermittent cash incentive system.

#### Intake

Interested individuals came to the lab and completed an eligibility questionnaire asking if they (a) were pregnant, (b) had asthma, (c) had an interest in increasing physical activity levels, or (d) had any reason why they should not be included. Eligible participants then completed the intake questionnaire, including demographic questions and participant's perceived fitness level. Participants received a Fitbit and were given instructions for use. Researchers demonstrated how to wear the Fitbit (shirt, pocket, or waist) and stated that it should be worn during all waking hours and that it was not waterproof. A schedule was set for the participant to come to the lab two to three times a week for ticket draws and to sync their Fitbit data (if needed).

#### Daily reporting

Participants texted, called, or emailed their step count at the end of each day (before midnight) to the experimenter. If the participant failed to initiate contact, the experimenter prompted the participant through a text or a phone call. During the intervention, the participant received immediate feedback from the experimenter: “You *did/did not* meet your goal today. Tomorrow, your goal is *x*.” Step counts were verified with raw data downloaded from the Fitbit website.

#### Ticket draws

Meeting a step goal during intervention earned the participant one ticket. Tickets were marked as either “winner” (50%) or “not a winner” (50%), and each ticket was also marked with a number that was associated with a particular amount of money. For example, 100 tickets were always in the bowl: 50 winners and 50 nonwinners. If the participant earned seven tickets for the week, they would choose seven tickets from the bowl in which they had the following chances of choosing tickets associated with the following cash values: $1.50 (small, 42%), $15 (medium, 5%), $25 (large, 2%), $50 (jumbo, 1%). After a ticket was drawn, the ticket was placed back in the bowl and the tickets were remixed to retain the same probabilities of winning. Participants came to the lab two to three times a week to draw tickets that had been earned based on meeting goals. A reference sheet for the number on the ticket was posted on the wall. Each time a participant pulled a ticket, it was logged with the date, participant number, and amount earned. As the participants did not come to the lab every day, multiple ticket draws could occur at one time. Participants chose whether to collect earnings as soon as they were earned or at the end of the experiment.

#### Establishing step criteria

During the intervention phase, researchers calculated step counts for ticket draws using a percentile schedule of reinforcement. We set *m* to seven prior observations. Research indicates that a *w* of 0.7 is a criterional probability of responding that capitalizes on extinction‐induced variability without resulting in ratio strain (Lamb et al., 2004). Based on Equation 1, using an *m* value of 7, and setting the probability at 0.7, yields *k* = (7 + 1) (1 – 0.7), or *k* = 2.4. We rounded *k* up to 3, which denotes the observation ranked third among the seven most recently observed, and ranked observations is the value the current observation must exceed to meet the criterion for reinforcement. Goals changed daily when the prior day's step count was incorporated into the *m* prior observations. No criterion exceeded 12,000 steps, as this was the maximum goal given to anyone.

#### Outtake

At the conclusion of the study, participants completed the exit questionnaire, which requested that participants provide demographic information and information about their physical activity levels. Participants also completed a treatment acceptability questionnaire, which asked participants to rate the acceptability of specific components of the intervention (liking, ease, and convenience), Fitbit, incentives, and technological concerns from 1 to 10 (see Supporting Information A). Participants also received any money they had yet to receive.

#### Data analysis

Table 1 shows summary statistics of the study population such as age, weight, height, body mass index, body fat percentage, and gender.

We calculated the number of days the Fitbit was worn, the number of days criteria were met, the number of days money was received, and the total money received for each participant. In addition, we calculated average step counts for each phase of the intervention and the average increase from baseline to intervention.

We used a repeated‐measures analysis of variance (ANOVA) to assess differences between mean step counts during the three phases of the study. All statistics were completed using α = .05. We also used visual analysis to assess changes in walking in the different phases of the intervention. A Pearson correlation coefficient was calculated to evaluate the correspondence between goal criteria during the intervention and step counts during the intervention.

We categorized participants into groups of high responders (meeting greater than 70% of goals and either a positive correlation or no statistically significant correlation between step counts and goals met, with a standard deviation less than 2,500 steps), mid responders (meeting either greater than 70% of goals or 50%–70% of goals while also having no statistically significant correlation between step counts and goals met, with a standard deviation greater than 2,500 steps), or low responders (meeting either 50%–70% of goals or less than 50% of goals while also having a negative but not a statistically significant correlation between step counts and goals met, with a standard deviation greater than 2,500 steps).

## RESULTS

Table 1 shows the 21 participants who completed the study and an additional 19 participants who were excluded because they walked more than 10,000 steps on average during the first baseline or were sick. Of the 21 participants who completed the study, 75% were female and had a mean age of 27.7 (*SD* = 12.9) years.

**FIGURE 1:**
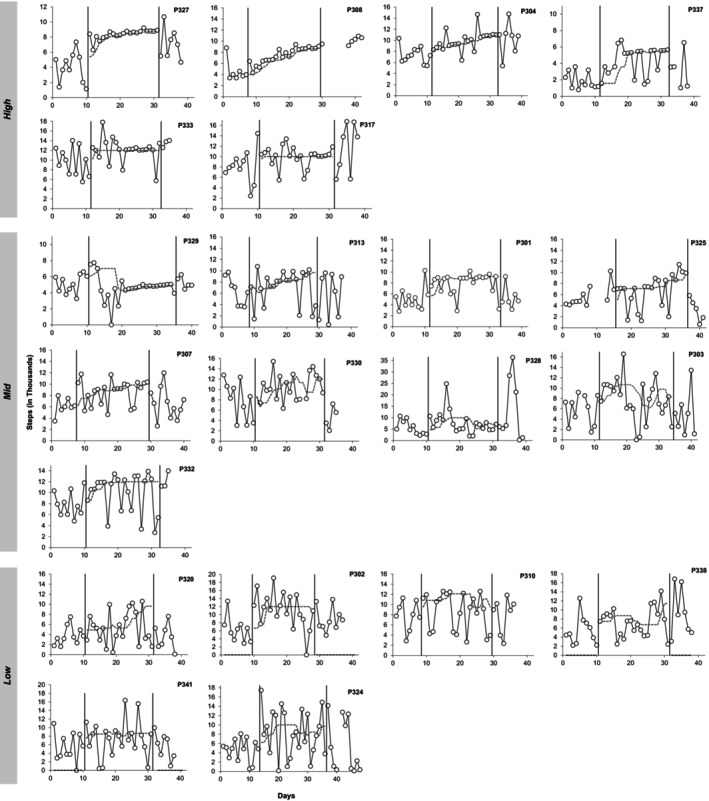
Daily step counts across conditions. Participants are grouped based on high, mid, and low responders. Open symbols indicate daily step counts, and the dark gray dashed line indicates daily goals.

Figure 1 shows individual participants' daily step counts ordered by high, mid, or low responders. Table 2 presents mean step counts along with standard deviations and percentage of goals met for each phase of the study; correlation coefficients between steps and goals during the intervention phase for each participant are also presented in Table 2. High responders all increased their steps during intervention relative to baseline. P327, P308, and P304's step counts systematically correlated with their goals; as goals increased, their step counts increased over the course of the intervention phase. Mid responders increased their steps relative to baseline conditions; however, they missed 35% of their goals. In addition, P329's mean step counts decreased from 5,129 steps during baseline to 4,631 steps during intervention. Data for P329 were affected by a dip at the start of the intervention phase; by the end of the phase, responding returned to be within baseline range. Mid responders did not show a statistically significant positive correlation between step goals and step counts. Low responders also increased their steps relative to baseline, but they missed 47% of their goals. In addition, mid and low responders demonstrated negative correlations between step goals and step counts, suggesting no consistent relation between goals and step counts.

**TABLE 2:** Step counts, goals met, correlation coefficients, and money earned.

	Step count								
Participant	BL1 (*M*)	BL1 (*SD*)	Int (*M*)	Int (*SD*)	BL2 (*M*)	BL2 (*SD*)	Goals met (%)	Correlation coefficient	Money earned ($)
**High responders**									
327	4,018.60	1,914.30	8,389.62	602.24	7,099.00	1,930.20	100	0.71*	63
308	4,546.00	1,774.50	7,480.23	1,366.30	9,970.40	606.20	100	0.92*	36
304	7,378.00	1,426.56	9,935.81	1,724.33	10,309.71	2,698.33	86	0.43	30
337	1,904.73	952.90	4,397.80	1,672.80	3,188.20	1,997.90	85	0.24	9
333	9,714.27	2,774.00	12,062.24	2,375.00	13,470.33	650.40	81	−0.05	46.50
317	8,133.20	3,128.20	10,029.95	1,893.90	11,531.14	4,506.60	76	−0.05	67
**Mid responders**									
329	5,129.30	1,091.30	4,630.76	1,524.30	5,253.80	642.60	72	−0.13	73
313	6,328.38	2,304.30	7,172.90	2,837.60	5,804.75	3,722.80	71	−0.08	31.50
325	5,673.73	1,800.60	6,796.48	2,904.70	3,999.71	2,082.60	71	0.35	15
301	5,143.36	2,081.68	7,922.77	1,674.47	5,328.83	2,255.52	68	−0.07	4.50
307	6,072.86	1,352.30	8,631.14	2,026.80	6,571.27	2,824.40	68	−0.02	52.50
330	7,924.70	3,533.70	10,581.00	2,519.90	4,588.25	1,955.90	67	0.004	10.50
328	5,656.60	2,908.70	7,792.52	4,763.60	13,161.25	12,739.60	57	0.02	39
303	5,795.10	2,704.69	7,772.39	3,873.35	5,029.86	3,615.65	57	0.02	75.50
332	7,967.90	2,194.50	9,946.27	3,369.80	12,130.33	1,308.00	55	−0.05	37.50
**Low responders**									
320	3,790.10	1,777.90	5,289.57	3,115.70	3,601.00	2,352.10	52	−0.10	13.50
302	6,327.44	3,005.12	11,196.21	4,296.79	8,897.00	2,819.37	65	−0.16	22.50
310	7,819.88	2,672.20	8,588.76	3,555.70	8,036.57	3,248.10	43	−0.18	83
338	5,353.60	3,039.60	7,296.43	3,047.70	9,324.71	4,989.40	45	−0.21	31
341	5,502.00	3,169.00	7,421.52	4,088.20	5,666.57	2,857.50	33	−0.24	9
324	4,639.23	2,289.60	8,267.09	4,815.30	5,368.55	5,466.90	43	−0.27	4.50
***M***	**5,943.76**	**2,280.75**	**8,171.50**	**2,764.21**	**7,539.58**	**3,108.10**	**66**	**0.41** *	**36**

*Note*: BL1 = Baseline 1; Int = intervention; BL2 = Baseline 2.

* *p* < .05.

**FIGURE 2:**
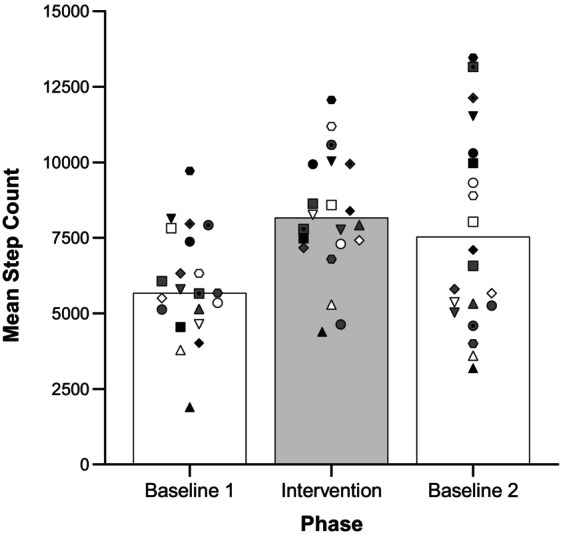
Mean step counts across conditions. Participants are grouped based on high (closed symbols), mid (gray symbols), and low (open symbols) responders across the three phases. Each symbol represents one participant's step counts during the three phases.

Table 2 and Figure 2 show participants' step counts during the study. Overall, participants substantially increased their step counts during the intervention phase. Mean step counts were 5,944 (*SD* = 1,778) during the initial baseline phase. Steps increased to a mean of 8,171 (*SD* = 2,005) during the intervention phase. During the return to baseline phase, the mean step count was 7,540 (*SD* = 3218). A repeated‐measures ANOVA revealed a significant difference between the three phases of the intervention, *F*(2, 40) = 11.49, *p* < .001. Simple contrasts revealed a difference between the first baseline and intervention phases, *t*(1, 20) = 8.55, *p <* .001, and a significant difference between BL1 and BL2, *t*(1, 20) = 2.83, *p* = .01, suggesting that the effects of the intervention persisted into BL2. There was no significant difference between the intervention and BL2, *t*(1, 20) = −1.15, *p* = .26. There was a significant correlation between steps and goals for the duration of the intervention, *r*(1,446) = 0.41, *p* < .001, suggesting that as goals increased, so did participant step counts. The percentage of goals met per participant during the intervention was 66% (*SD* = 18%). The mean amount of money earned was $35.90, with the amount for all participants totaling $754.

### Treatment acceptability

Overall, participants rated the intervention favorably, *M* = 9.4 (*SD* = 0.9) and indicated that it helped increase their physical activity, *M* = 7.7 (*SD* = 2.4). Participants also indicated that the program was relatively convenient and effective, *M* = 8.3 (*SD* = 0.7). Ratings of the Fitbit were also positive, *M* = 8.8 (*SD* = 0.4). Finally, participants indicated that they liked getting daily step goals, *M* = 8.6 (*SD* = 0.7). Prize draws were rated positively (*M* = 9.4, *SD* = 0.2), and participants did not have many concerns about the use of technology, *M* = 0.5 (*SD* = 1.1).

## DISCUSSION

The present study extended the intermittent incentives literature to include an evaluation of the efficacy of cash incentives for increasing daily step counts. Overall, the intervention was effective, with participants increasing their step counts by 73%. Participants met an average of 66% of their goals during the intervention and earned a mean of $35.90 throughout the intervention. In addition, 67% (*n* = 14) of 21 participants were classified as high or mid responders based on our criteria.

Our findings are consistent with those of research concerning intermittent incentives in addiction (e.g., Petry et al., 2000, 2005, 2006). The cash incentives in the present study systematically increased step counts during the intervention phase above BL1. Other studies have similarly demonstrated that intermittent cash incentives can foster dietary self‐monitoring and weekly weight loss (Voils et al., 2021) and adherence to daily self‐monitoring of stress and accelerometer use (Husain et al., 2019). A study from the addiction literature suggests there may be greater effects when cash incentives rather than vouchers or prizes are used (Vandrey et al., 2007). The current study did not experimentally compare cash incentives with vouchers or tangible prizes, but future research should evaluate a direct comparison between intermittent cash incentives and prizes.

Intermittent incentives for physical activity have yielded mixed effects. Several studies have demonstrated significant improvement in physical activity when incentives were provided relative to when they were not (May & Treadwell, 2020; Patel et al., 2018). The participants in the current study performed comparably to those in these studies, where most improved their responses (e.g., May & Treadwell, 2020; Patel et al., 2018; Petry et al., 2000). However, Washington et al. (2014) reported a smaller effect, with only four out of 11 individuals improving their step counts. Two methodological differences between the present study and Washington et al. may account for the different results. The present study excluded participants whose mean step counts during baseline exceeded 10,000 steps, eliminating any ceiling effect that may have been present in Washington et al. For example, two of the participants in Washington et al. had mean step counts above 10,000, leaving them with less room for improvement. In addition, the present study used cash incentives rather than the prizes available in the Washington et al. study. It is possible that prizes were a less effective incentive than the cash incentives offered in the current study (Vandrey et al., 2007).

Intermittent incentives can be less expensive than continuous incentives contingent on the same behavior (e.g., Petry & Martin, 2002; Petry et al., 2000). The present study cost a total of $754 throughout a 3‐week intervention period for 21 subjects, costing a total of $35.90 per participant. In addition, the intermittent nature of the incentives may increase the intervention's generalizability (Stokes & Baer, 1977) and resistance to extinction postintervention (Craig, 2023) by reducing the participants' ability to discriminate between incentivized conditions (intervention) and nonincentivized conditions (BL2). The present study demonstrated persistent effects of the intervention in the return to baseline phase such that the mean step count was significantly higher than that in the first baseline and not different from that in the intervention phase. This nonreversal limits the demonstration of functional control; the presence of the independent variable (incentives) may not have been the only variable causing an increase in step counts. However, this is consistent with literature concerning resistance to extinction that demonstrates the persistent effects of the intermittent reinforcement during extinction conditions (Craig, 2023). Future research should investigate longer postintervention follow‐ups to determine whether the effect is persistent over longer durations.

There are several limitations of the present study that require further study. First, restricting the participants from using the Fitbit application or website required setting goals based on participant reports of their step counts until verification was conducted in the office. There were a few instances where a participant misreported or could not access their step count if it was after midnight. In addition, researchers attempted to contact the participant as close to midnight as possible, but participants could have reported step counts and then walked additional unrecorded and unreported steps before the end of the day. There were also nine participants that withdrew before the intervention phase due to sickness, technical problems, or other factors. In addition, the intermittent and probability‐based nature of the incentives meant that some participants earned high amounts, whereas some earned low amounts during the intervention. For some participants, the low, probabilistic incentives may not have been enough to increase step counts. The lowest amount earned throughout the study was $4.50, which was earned by two participants, P301 and P324. P301 met 68% of their goals and performed consistently well, but P324 met only 43% of their goals. Tailoring the probability and incentive values to each individual based on a measure of sensitivity to probabilistic rewards (e.g., probability discounting; Ghitza et al., 2013; Rachlin et al., 1991) may increase efficacy. In addition, participants could choose when they collected their earned incentives and the longer delays in collecting earnings may have affected the efficacy of the incentives (Packer et al., 2012).

The present study extended the intermittent incentives literature in demonstrating the efficacy of intermittent cash incentives. Strengths of this study include the intermittent nature of the reinforcement, the use of cash instead of prizes, and the application of this method to physical activity. These findings support prior research demonstrating the efficacy of intermittent reinforcement of other behaviors (e.g., substance use). Overall, intermittent incentives reduce the cost of the intervention and may increase its generality to naturalistic settings.

## CONFLICT OF INTEREST STATEMENT

The authors have no conflicts of interest to disclose with the current manuscript.

## ETHICS APPROVAL

This study received institutional review board approval and was conducted in accordance with established ethical guidelines for human participants.

## Supporting information


**Data S1:** Supporting Information.

## Data Availability

The treatment acceptability questionnaire can be found in supporting information. Data are available upon request.
