# Supplementary material for: Effectiveness of intermittent cash incentives to increase step counts
Source: J Appl Behav Anal. 2024 Dec 27;58(1):81–90. doi: 10.1002/jaba.2929 (PMC11803350; doi:10.1002/jaba.2929)
Supplement: Supplementary file 1 — Data S1: Supporting Information. [file JABA-58-81-s001.pdf]

Supporting Information

Supporting Information A

Treatment Acceptability Questionnaire

Circle the rating you think for each question based on your experience during the study.

Overall Intervention

1. How easy to use was the physical activity program that you completed?

   0 1 2 3 4 5 6 7 8 9 10

   Not easy Very easy

2. How helpful was the program in increasing your physical activity?

   0 1 2 3 4 5 6 7 8 9 10

   Not helpful Very helpful

3. How convenient was the physical activity program that you just completed?

   0 1 2 3 4 5 6 7 8 9 10

   Not convenient Very convenient

4. How effective was the physical activity program that you just completed?

   0 1 2 3 4 5 6 7 8 9 10

   Not effective Very effective

5. How fair was the physical activity program that you just completed?

   0 1 2 3 4 5 6 7 8 9 10

   Not fair Very fair

6. How flexible was the physical activity program that you just completed?

0 1 2 3 4 5 6 7 8 9 10

Not flexible Very flexible

Fitbit

7. How much did you like using the Fitbit to monitor your physical activity?

0 1 2 3 4 5 6 7 8 9 10

Not at all Very much

8. How easy was it to use the Fitbit to monitor physical activity?

0 1 2 3 4 5 6 7 8 9 10

Not easy Very easy

9. How convenient was the Fitbit in monitoring your physical activity?

0 1 2 3 4 5 6 7 8 9 10

Not convenient Very convenient

10. How effective was the Fitbit in monitoring your physical activity?

0 1 2 3 4 5 6 7 8 9 10

Not effective Very effective

Goals

11. How much did you like getting daily step count goals?

0 1 2 3 4 5 6 7 8 9 10

Not at all Very much

12. How effective do you think the goals were in changing your physical activity?

0 1 2 3 4 5 6 7 8 9 10

Not effective Very effective

13. How easy was reporting step counts to researchers each day?

0 1 2 3 4 5 6 7 8 9 10

Not easy Very easy

Incentives

14. How much did you like earning monetary prize draws based on your physical activity?

0 1 2 3 4 5 6 7 8 9 10

Not at all Very much

15. How helpful was earning monetary prize draws based on your physical activity?

0 1 2 3 4 5 6 7 8 9 10

Not helpful Very helpful

Technology

16. To what extent were you concerned about electronic information (for example, privacy issues, sending information over the internet, texting, uploading physical activity data) as part of the program?

0 1 2 3 4 5 6 7 8 9 10

Not concerned Very concerned
